# High-grade cervical intraepithelial neoplasia in human papillomavirus self-sampling of screening non-attenders

**DOI:** 10.1038/bjc.2017.371

**Published:** 2017-11-14

**Authors:** J U H Lam, K M Elfström, D M Ejegod, H Pedersen, C Rygaard, M Rebolj, E Lynge, K E Juul, S K Kjær, J Dillner, J Bonde

**Affiliations:** 1 Department of Pathology, Copenhagen University Hospital Hvidovre, Kettegård Allé 30, 2650 Hvidovre, Copenhagen, Denmark; 2 Department of Laboratory Medicine, Karolinska Institutet, Stockholm 14186, Sweden; 3 Clinical Research Centre, Copenhagen University Hospital Hvidovre, Kettegård Allé 30, 2650 Hvidovre, Copenhagen, Denmark; 4 Department of Public Health, University of Copenhagen, Copenhagen 1014, Denmark; 5 Unit of Virus, Lifestyle and Genes, Danish Cancer Society Research Center, Copenhagen 2100, Denmark; 6 Department of Obstetrics and Gynecology, Copenhagen University Hospital Rigshospitalet, Copenhagen 2100, Denmark; 7 Department of Medical Epidemiology and Biostatistics, Karolinska Institutet, Stockholm 14186, Sweden

**Keywords:** HPV self-sampling, non-attenders, cervical cancer screening, HPV primary screening, cervical intraepithelial neoplasia, implementation, Denmark

## Abstract

**Background:**

Self-sampling for human papillomavirus (HPV) offered to women who do not participate in cervical cancer screening is an increasingly popular method to increase screening coverage. The rationale behind self-sampling is that unscreened women harbour a high proportion of undetected precancer lesions. Here, we compare the cervical intraepithelial neoplasia grade 2 or worse (⩾CIN2) detection rate between non-attenders who participated in self-sampling and women attending routine screening.

**Methods:**

A total of 23 632 women who were qualified as non-attenders in the Copenhagen Region were invited for HPV-based self-sampling. Of these, 4824 women returned a self-sample, and HPV-positive women were referred for cytology and HPV co-testing as follow-up. The entire cohort and a reference cohort (3347 routinely screened women) were followed for histopathology confirmed ⩾CIN2. Odds ratio (OR) and the relative positive predictive value of ⩾CIN2 detection between the two populations were estimated.

**Results:**

Women participating in self-sampling had a higher ⩾CIN2 detection than women undergoing routine cytology-based screening (OR=1.83, 95% CI: 1.21–2.77) and a similar detection as routinely screened women tested with cytology and HPV testing (OR=1.03, 95% CI: 0.75–1.40). The positive predictive value for ⩾CIN2 was higher in screening non-attenders than in routinely HPV- and cytology-screened screened women (36.5% *vs* 25.6%, respectively).

**Conclusions:**

Self-sampling offered to non-attenders showed higher detection rates for ⩾CIN2 than routine cytology-based screening, and similar detection rates as HPV and cytology co-testing. This reinforces the importance of self-sampling for screening non-attenders in organised cervical cancer screening.

A major challenge in preventing cervical cancer is the large proportion of women who are screening non-attenders. In the organised Danish cervical cancer screening programme, women aged 23–49 years are invited every 3 years for screening, and women 50–65 years of age are invited every 5 years. Approximately 75% of the target population is screened following these recommendations, with minor regional and annual fluctuations (Danish Quality Assurance Database for the Cervical Cancer Screening Program). Yet, 45% of new cancer cases are diagnosed among non-attenders (Dugue *et al*, 2012).

Human papillomavirus (HPV)-based self-sampling has been shown to increase screening participation in several studies (Gok *et al*, 2010; Enerly *et al*, 2016; Sultana *et al*, 2016). In May 2014, we initiated ‘The Copenhagen Self-sampling Initiative’ (CSi) pilot to gain experience in how to best offer non-attenders in the Capital Region of Denmark an HPV self-sampling test as an alternative to the standard physician-taken cytology sample. The CSi was designed as an implementation opt-in pilot study, where approximately half of the women residing in the Capital Region who had missed at minimum one screening round were invited to order a self-sampling brush. As the pilot implementation was population based, the invited women had variable screening history, ranging from those last screened 4 years before the self-sampling invitation to those who had never been screened. Overall, ∼20% of the 23 632 invited women participated by returning a self-sampling brush. Of these, 40% were long-term unscreened with no registered cytology in ⩾10 years (Lam *et al*, 2017). By November 2016, an additional 18% of the invited women passively or actively declined the self-sampling invitation and instead went to their own general practitioner (GP) for screening. In total, 38% of all invited women were screened in an 18-month period after receiving the self-sampling invitation.

A successful self-sampling strategy should not be less sensitive for cervical intraepithelial neoplasia grade 2 or worse (⩾CIN2) detection than routine screening. We investigated the detection rate of ⩾CIN2 among the screened women in a real-life self-sampling setting, the CSi, and estimated the positive predictive value of this procedure (with ⩾CIN2 detection as a threshold). Furthermore, we compared the observations to a population-based routine screening cohort from the Horizon study (Preisler *et al*, 2013, 2016; Rebolj *et al*, 2013, 2014a, b, 2015a, 2016a, b; Bonde *et al*, 2014; Ejegod *et al*, 2015). Samples from both cohorts were tested in the same laboratory.

## Materials and methods

**Figure 1:**
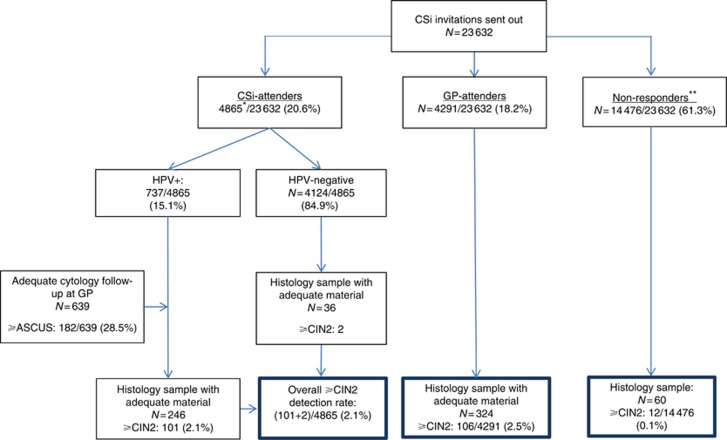
**Flowchart of the CSi study design, follow-up triage, and ⩾CIN2 detection rate.** *Of all tested samples, 4 were invalid on all HPV assays, 773 samples had a positive test result on any of the three assays, and 246 of these had biopsies taken. Of the 4124 HPV-negative women, 36 had a biopsy taken (for an unknown reason). Because of a hardware failure on the Onclarity assay, some women received their result based on CLART and HC2 only. The samples were later re-tested on Onclarity, with 36 women having a positive test result. Owing to hospital practice, these women did not receive a supplement result. The flowchart illustrates the follow-up process based on the result the women received, that is, the result that influenced their clinical management. **Non-responders remained unscreened during the whole study period; the CIN2 or worse cases detected might be symptom-related indications rather than screening. CIN=cervical intraepithelial neoplasia.

The 23 632 screening non-attenders in the CSi implementation study were invited to participate in screening via self-sampling using an Evalyn brush (Rovers, Oss, The Netherlands). Non-attenders were defined as women who had not been screened for at least 4 (if aged 27–49 years) or 6 years (if aged 50–65 years). Invitations for self-sampling were sent in batches of 1000 women from May 2014 to April 2015; during this time, new routine screening invitations may have been sent from the screening programme. The women invited for self-sampling were sent a letter with information on cervical cancer screening and the association between HPV and cervical cancer; they could order a self-sampling brush from our laboratory and return it using a prestamped envelope. In total, 4865 returned their self-sampling test for HPV testing on two or three different HPV assays (Figure 1). An additional 4291 women were not screened via self-sampling, but screened by a GP after receiving the self-sampling invitation letter. Note that these numbers differ from those reported previously (Lam *et al*, 2017), owing to a longer follow-up (November 2016 instead of December 2015). The HPV positivity was determined on the CLART (Genomica, Madrid, Spain) and Onclarity (BD, Sparks, MD, USA) assays for all samples. A subset of the first 1008 samples was also analysed on Hybrid Capture 2 (HC2; Qiagen, Hilden, Germany). Upon a positive HPV self-sample (i.e., if any of the three HPV assays gave a positive test result), women were referred to a GP-taken cytology follow-up sample. The follow-up samples were co-tested with HPV and cytology. Based upon the outcome of this follow-up test result, women were referred to re-testing or gynaecology examinations. For the current study, the women included in CSi were followed for 18 months (until November 2016) after the last invitations were sent.

**Figure 2:**
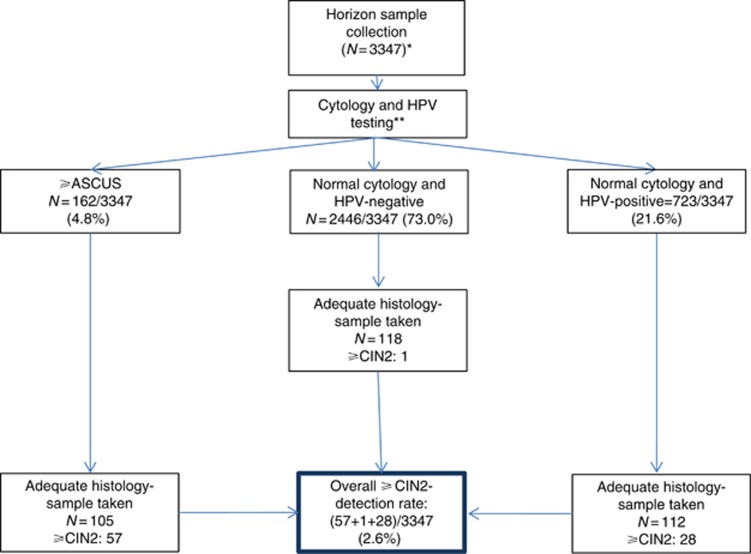
**Flowchart of the Horizon study design, follow-up triage, and ⩾CIN2 detection.** *Of the 3347 screened women, 16 (0.48%) were inadequate for cytology reading. One of the women had a histology sample registered with normal findings. This contributed to a total of 336 biopsies taken. **Women were followed-up according to routine practice based on the cytology result only. An active follow-up was performed on women with cytology normal HPV-positive findings according to the study protocol that was repeat testing after 18 months. Women with normal cytology and negative HPV test results were referred back to the routine screening programme; some of these had a histology sample taken despite the recommendations. Women who were cytology negative and HPV positive were invited for repeated testing after 18 months. All HPV test results reported here took into account the testing results on any of the four assays. CIN=cervical intraepithelial neoplasia.

For comparison, we have used data from the Horizon study that was a population-based study of women routinely screened at their GP. It included residual material from 5034 consecutive SurePath cytology samples from women aged 16–89 years. The samples were additionally tested for HPV using four different HPV assays (HC2, cobas, CLART, and APTIMA), as described previously (Preisler *et al*, 2013, 2016; Rebolj *et al*, 2013, 2014a, b, 2015a, 2016a, b; Bonde *et al*, 2014; Ejegod *et al*, 2015). Horizon was nested into routine practice. The women were therefore triaged according to routine practice if they had cytology abnormal findings (defined as atypical squamous cells of undetermined significance, ⩾ASCUS). As an extra intervention in the Horizon study, cytology normal and HPV-positive women had an active follow-up according to the study protocol (Figure 2), where these women were invited for repeat testing after 18 months.

Both studies were conducted at the Department of Pathology, Copenhagen University Hospital (Supplementary Table 1). The department was responsible for sending screening invitations and testing of all cervical samples in the Copenhagen and Frederiksberg municipalities when the Horizon study was undertaken between June and August 2011 (this area covered ∼15% of all Danish women eligible for cervical screening). From 2012 onwards, the department was responsible for sending invitations and testing all samples from the whole Capital Region (∼33% of all eligible women), where the CSi was implemented between May 2014 and November 2015.

In order to ensure comparability of the cohorts, we excluded women from the Horizon study who were outside the self-sampling age range of 27–65 years (*n*=1040), as well as women with referral samples (which was women who had ASCUS or more severe abnormality diagnosis registered in the previous 15 months, or were HPV positive in the past 12 months, *n*=647). Finally, the two populations, Horizon (*n*=3347) and CSi (*n*=23 632), were included in the analysis.

### Data sources

Women’s screening status and history were retrieved from the National Pathology Database (Patobank). Patobank is a nationwide registry that covers all specimens from all pathology departments in Denmark, and has been highly complete since mid-2000s (Bjerregaard and Larsen, 2011). Women’s screening status was determined by calculating the time between the most recent test on record between 1 January 2000 and 6 May 2014, when we retrieved the list of eligible women to be invited for self-sampling. For the Horizon study, this was between 1 January 2000 and the date the Horizon study sample was collected in the routine laboratory.

If the referral, GP-taken, follow-up sample was cytology normal and HPV positive, women were recommended for retesting after 12 months. The data for the CSi study were retrieved 18 months after the last invitations were sent out, in November 2016. In the Horizon study, cytology-normal and HPV-positive women were invited by the laboratory to be retested after 18 months. The histology follow-up took place until December 2013 that was ∼30 months after study start.

### Statistical analysis

Means and proportions were used to describe the study populations. The outcome of interest was detection of CIN2 or worse (⩾CIN2) *vs* normal biopsy/CIN1/no biopsy. All abnormal histological findings in the CSi study were confirmed by a chief physician reviewing the description, diagnosis code, and electronic medical records. Odds ratios (ORs), their 95% confidence intervals (CIs), and *P-*values of the association between ⩾CIN2 detection and explanatory variables were estimated in a logistic regression model. The ORs were reported as crude and adjusted, where age, study and the women’s screening history were taken into account. Cofactors were examined separately and as potential confounders. Age and screening history were determined to be a confounder of the association between the study population, the exposure of interest, and ⩾CIN2 detection, and were therefore included in the adjusted model.

Four mutually exclusive population types were defined for the analysis: one for the participants in the Horizon study, and three for the women included in the CSi study: CSi-attenders (women who returned their self-sampling test), GP-attenders (women who had a physician-taken cytology sample), and non-responders (invited women who neither responded to the CSi invitation nor were screened by a GP).

Positive predictive value of ⩾CIN2 detection was calculated as the proportion of ⩾CIN2 cases found among those who had an adequate biopsy taken.

Screening history was categorised into two groups: intermittently screened in case the women’s latest screening samples were registered within 10 years before the study, and long-term unscreened in case where the women’s last screening samples, if any, were registered ⩾10 years before the study.

All statistical analyses were performed using Stata 13.1. (StataCorp, College Station, TX, USA).

### Ethical approvals

Linkage of the data for both CSi and Horizon was approved by the Danish Data Protection Agency under notification number AHH-2015-084 I-Suite number: 04139, and AHH-2015-084, I-Suite number: 04139, respectively. HPV testing in the Horizon study did not require ethical approval, as it was undertaken as a quality development study in concordance with the Committee Act under The National Committee on Health Research Ethics (2011 §14, part 3); the 18-month follow-up round was approved by the Ethics Committee of the Danish Capital Region (journal no. H-4-2012-120), and the women provided informed consent. The CSi was a pilot implementation, mandated by the Danish Health Authority and the Capital Region of Denmark; ethical approval was therefore not required. Women who ordered the self-sampling kit implicitly agreed to participate in the implementation.

## Results

Of the 23 632 women invited to participate in self-sampling, 4865 (20.6%) returned a self-sampling test, typically within the first 6 months, 4291 (18.2%) had a cytology sample taken by a GP, and 14 476 (61.3%) had neither within the register-based follow-up period (Figure 1). Of the 4865 CSi-attenders (women who returned a self-sampling test), 737 (15.1%) were HPV positive, and referred for an immediate routine cytology sample with HPV co-testing. In total, 639 (86.8%) women had cytology and HPV follow-up, among whom 98 ⩾CIN2 were detected. Six HPV-positive women instead opted for a direct colposcopy, and three of these had ⩾CIN2. Two additional ⩾CIN2 were detected among HPV-negative CSI-attenders, despite not being recommended for additional testing. In total, therefore, 103 (2.1%) ⩾CIN2 were detected among 4865 CSi-attenders (Table 1). Among the 4291 GP-attenders, 106 (2.5%) had a diagnosis of ⩾CIN2, whereas 12 out of 14 476 (0.1%) women from the non-responders group had a ⩾CIN2 detected; the reason for these biopsies is unknown, but may have been taken in response to symptoms – six of these lesions were cervical cancer (Table 1).

In the Horizon study (Figure 2), 3347 screened women were offered follow-up in case they had abnormal cytology or a positive HPV test result on any of the four assays. The remaining women were referred back to the routine screening programme. Overall, 336 histology diagnoses were retrieved with 86 ⩾CIN2 (2.6%) in the Horizon study. When excluding the ⩾CIN2 found among women in the per-protocol arm (repeat testing for women cytology normal and HPV positive), and only including the ⩾CIN2 found in the cytology stand-alone protocol (which reflects the routine screening programme), the ⩾CIN2 detection was 58/3347 (1.7%).

The CSi-attenders were on average 7 years older than GP-attenders in CSi and in the Horizon study (mean ages: 47.1 *vs* 40.3 *vs* 40.6 years, respectively). Almost all (94.2%) women in the Horizon study were screened at least once in the preceding 10 years. This was the case for approximately two-thirds of CSi-attenders (60.1%) and GP-attenders (69.2%), but for only 36.0% of the CSi non-responders (who were also non-responders to routine screening). Among the adequate biopsies, CSi-attenders and GP-attenders had a higher detection of ⩾CIN2 (positive predictive value of an adequate biopsy: 36.5% and 32.7%, respectively) than non-responders (20.0%) and women included in the Horizon study (25.6%). Among all detected ⩾CIN2, women were slightly more likely to have ⩾CIN3 detected if they were CSI-attenders (78.6% of all ⩾CIN2 diagnoses were ⩾CIN3) or GP-attenders (78.3%) than when they were routinely screened (72.1%, Horizon). In total, 18 women were diagnosed with cervical cancer in the CSi study, and one in the Horizon study.

Women aged >30 years were significantly less likely to have a ⩾CIN2 diagnosis (*P*<0.01) compared with women aged 27–29 years, and this did not change after adjustment for population type and screening history (Table 2). Without any adjustment, intermittently screened women (women who were screened within the past 10 years) appeared to have a higher risk of a ⩾CIN2 diagnosis than long-term unscreened women (*P*<0.01). After adjustment for age and population type, no significant difference was observed between intermittently and long-term unscreened women (*P*=0.97). CSi-attenders (*P*=0.18) and GP-attenders (*P*=0.78) appeared to have a similar (crude) risk of ⩾CIN2 detection as women screened in the Horizon study that remained virtually unchanged after adjustment for age and screening history (*P*=0.88 and *P*=0.81, respectively). This was also the case if screening history was re-defined as either the number of unscreened years or dichotomised as unscreened for <6 and ⩾6 years (*P*= 0.40 and *P*=0.78, respectively; data not reported). When we excluded the effect of HPV-based screening in the Horizon study and compared the ⩾CIN2 detection in CSi only with cytology-based detection in Horizon (i.e., routine cytology-based screening), CSi-attenders had a 1.83 times higher detection of ⩾CIN2 (adjusted OR=1.83, 95% CI: 1.21–2.77, *P*<0.01). The CSi non-responders had a significantly lower detection of ⩾CIN2 than routinely screened women (*P*<0.01).

## Discussion

In our population-based study of screening non-attenders, HPV-based self-sampling detected a similar proportion of ⩾CIN2 as routine screening with cytology and HPV-based co-testing, and a higher proportion than stand-alone routine cytology-based screening. Furthermore, a slightly higher proportion of CIN3 and cervical cancer was also observed among the CSi-attenders. The positive predictive value for ⩾CIN2 of a biopsy was slightly higher in CSi compared with Horizon. This indicated that self-sampling was effective and efficient in detecting high-grade CIN lesions whose treatment might prevent future cases of cervical cancer.

From an implementation point of view, the CSi and the Horizon studies illustrate the true impact of screening activities in a real-world setup. The ⩾CIN2 rate found in the CSi therefore demonstrated the expected rates that would be found if self-sampling would be added as an additional screening option for women not attending routine screening.

Women who remained unscreened even after a self-sampling invitation were highly unlikely to have a ⩾CIN2 lesion detected quite simply as they were not examined. Most of their CIN2 or CIN3 lesions would go undetected until overtly symptomatic. In our implementation study, 10% of all ⩾CIN2 diagnoses in these women were cancer, compared with ⩽2% in women undergoing routine or self-sampling screening. These women remain a research priority to determine a better way of attracting them to screening, although in younger birth cohorts this need will be attenuated owing to HPV vaccination.

Few studies have compared the ⩾CIN2 detection in screening non-attenders who participate in self-sampling and in routinely screened women. Bais *et al* (2007) compared 736 women undergoing self-sampling (corresponding to our ‘CSi-attenders’ group) and an age-matched population-based cohort of 6208 routinely screened women (corresponding to the ‘Horizon’ population) as the reference group. This study found an overall higher ⩾CIN2 detection in the self-sampling group compared with routinely screened women (OR=2.59, 95% CI: 1.31–5.12). A similarly higher detection of ⩾CIN2 was found for self-sampling participants compared with routine screening participants by Gok *et al* (2012), with a relative risk of 1.63 (95% CI: 1.4–1.9). Sultana *et al* (2016), on the other hand, reported in an opt-out study that the detection of ⩾CIN2 in 1649 women who returned a self-sampling swab and in all screened women aged 30–69 years from the Australian state of Victoria were insignificantly different from each other. These findings could be explained by a shorter follow-up period in the self-sampling study (6 months) that might lower the number of ⩾CIN2 detected.

Our study had important strengths. Both the CSi and the Horizon studies were population based and included representative samples of routinely screened and routinely unscreened women. With the use of the national Patobank for identifying outcomes in both studies, our analysis had high data completeness in identifying the detected ⩾CIN2 cases. This was the case even if the women moved outside of the laboratory’s catchment area, thereby avoiding information bias. However, some limitations with regard to the comparability of the CSi and the Horizon studies need to be mentioned. Some of the CSi-attenders (N=41) and GP-attenders (N=1029) in the CSi study had a follow-up time shorter than 1.5 years because they were screened very late; these women may have had ⩾CIN2 detected after the date of data retrieval. The two populations differed somewhat in terms of their respective screening catchment areas. The Horizon study covered the most urbanised parts of the Capital Region, whereas the CSi covered the whole Capital Region. Furthermore, the two studies varied in their design, HPV testing methods and triage procedures (Supplementary Table 1). Different HPV assays were used in the studies and the Horizon study showed that HPV detection depends on the assay (Rebolj *et al*, 2014b) that may affect the screening follow-up referrals (Rebolj *et al*, 2016a). For this analysis, all histological findings were retrieved, regardless of HPV results to ensure that all outcomes were captured. The CSi study was undertaken 3 years after the Horizon study. Changes in CIN incidence may occur over time; however, with only a few years between the studies, we consider it unlikely. No changes in ⩾CIN2 detection over a 10-year period were previously found in the catchment area of the Roskilde laboratory that is in close geographical proximity to Copenhagen (Rebolj *et al*, 2015b). Primary screening recommendations also remained unchanged during those 3 years.

Based upon the resulting detection of ⩾CIN2, self-sampling in this setting appears to be more effective than the current cytology routine screening, and would therefore lead to detection of an extra number of high-grade lesions requiring treatment. However, to finetune its implementation within an organised screening programme, several issues still need to be addressed. These include a definition of an optimal screening interval after a negative HPV self-sample that will be investigated by register-based monitoring of the screening and diagnostics activities of these women in the forthcoming years via the Patobank. Furthermore, we speculate that loss to follow-up could be reduced among self-sampling non-attenders if, for example, a direct referral to colposcopy is implemented for women with specific high-risk HPV genotypes replacing part of the current uniform referral for cytology follow-up. Finally, it is crucial for the integrity of an organised screening programme to foresee the proportions of women who may switch from GP-screening to self-sampling by skipping calls for screening in order to obtain a non-responder status (Rozemeijer *et al*, 2015). As organised cervical cancer screening depends on participation within regular intervals, the latter could over time develop into a challenge.

## Conclusion

We observed higher detection rates of ⩾CIN2 with HPV-based self-sampling than in the routine cytology-based screening, suggesting that self-sampling is a good screening alternative for non-attenders. Furthermore, the proportion of high-grade CIN lesions among all biopsies was high, suggesting that the chosen referral approach was efficient.

## Figures and Tables

**Table 1:** The characteristics of the two study populations

	**CSi (*****N*****=23** **632)**						**Horizon (*****N*****=3347)**	
	**CSi-attenders (*****n*****=4865)**		**GP-attenders (*****n*****=4291)**		**Non-responders (*****n*****=14** **476)**		**GP-taken sample (*****n*****=3347)**	
Mean age (years; 95% CI)	47.1	(46.8–47.5)	40.3	(40.0–40.6)	47.6	(47.4–47.8)	40.6	(40.3–41.0)
Age group (years)								
27–29	385	7.9%	588	13.7%	1318	9.1%	478	14.3%
30–39	1092	22.4%	1657	38.6%	2962	20.5%	1295	38.7%
40–49	1215	25.0%	1311	30.6%	3107	21.5%	897	26.8%
50–59	1275	26.2%	514	12.0%	4099	28.3%	462	13.8%
60–65	898	18.5%	221	5.1%	2990	20.7%	215	6.4%
Total	4865	100.0%	4291	100.0%	14 476	100.0%	3347	100.0%
Screening history^a^								
Long-term unscreened	1621	39.9%	927	30.8%	7540	64.0%	133	5.8%
Intermittently screened	2440	60.1%	2083	69.2%	4243	36.0%	2158	94.2%
Total	4061	100.0%	3010	100.0%	11 783	100.0%	2291	100.0%
Histology (regardless of the HPV test result)								
Normal	134	47.3%	188	57.8%	47	78.3%	206	56.3%
CIN1	45	15.9%	30	9.2%	1	1.7%	44	12.0%
CIN2	22	7.8%	23	7.1%	0	0.0%	24	6.6%
CIN3	76	26.9%	76	23.4%	6	10.0%	61	16.7%
Cervical cancer	5	1.8%	7	2.2%	6	10.0%	1	0.3%
Inadequate material	1	0.4%	1	0.3%	0	0.0%	30	8.2%
Total	283	100.0%	325	100.0%	60	100.0%	366	100.0%
⩾CIN2 detection (regardless of the HPV test result)								
<CIN2	179	63.5%	218	67.3%	48	80.0%	250	74.4%
⩾CIN2 (positive predictive value)	103	36.5%	106	32.7%	12	20.0%	86	25.6%
Total	282	100.0%	324	100.0%	60	100.0%	336	100.0%
Positive predictive value of ⩾CIN2 detection compared with Horizon	*P*<0.01		*P*=0.02		*P*=0.93		Reference	

Abbreviations: CI=confidence interval; CIN=cervical intraepithelial neoplasia; GP=general practitioner; HPV=human papillomavirus; Intermittently screened=cytology sample registered within the past 10 years (though not in the last screening round, see eligibility criteria); Long-term unscreened=no cytology sample registered in the past 10 years.

a Restricted to women aged ⩾34 years, *N*=21 145 for both studies combined. These women have been recommended for screening for at least 10 years.

**Table 2:** Odds ratios and 95% confidence intervals for ⩾CIN2 detection, by age, study population, and screening history

	**Crude OR**				**OR, mutually adjusted**			
	**OR**	**95% CI**		*P*	**OR**	**95% CI**		*P*
**Age group (years)**								
27–29	1 (Ref.)				1 (Ref.)			
30–39	0.59	0.44	0.78	<0.01	0.53	0.39	0.71	<0.01
40–49	0.32	0.23	0.45	<0.01	0.31	0.22	0.44	<0.01
50–59	0.20	0.13	0.29	<0.01	0.27	0.18	0.41	<0.01
60–65	0.13	0.07	0.22	<0.01	0.20	0.12	0.35	<0.01
**Population**								
Horizon (*N*=3347)^a^	1 (Ref.)				1 (Ref.)			
CSi-attenders (*n*=4865)	0.82	0.61	1.10	0.18	1.03	0.75	1.40	0.88
GP-attenders (*n*=4291)	0.96	0.72	1.28	0.78	0.96	0.71	1.30	0.81
Non-responders (*n*=14 476)	0.03	0.02	0.06	<0.01	0.04	0.02	0.07	<0.01
**Screening history**								
All women (*N*=26 979)								
Long-term unscreened	1 (Ref.)				1 (Ref.)			
Intermittently screened	2.02	1.59	2.57	<0.01	1.00	0.77	1.29	0.97
34–65 years (*N*=21 145)								
Long-term unscreened	1 (Ref.)				1 (Ref.)			
Intermittently screened	1.91	1.41	2.60	<0.01	0.86	0.62	1.20	0.38

Abbreviations: CI=confidence interval; CIN=cervical intraepithelial neoplasia; CSi-attenders=women invited to CSi who returned a self-taken sample; GP-attenders=women invited for CSi who were screened by a general practitioner; Intermittently screened=women with a cytology sample registered within the past 10 years; Long-term unscreened=women without a cytology sample registered in the past 10 years; Non-responders=women invited to CSi without a self-sampling or a GP cytology sample until end of follow-up; OR=odds ratio.

a When using the per-protocol arm (cytology stand-alone screening) from the Horizon study as the reference group, the adjusted odds ratio for ⩾CIN2 detection for CSi-attenders was OR=1.83 (95% CI: 1.21–2.77), *P*<0.01.
