# Supplementary material for: High-grade cervical intraepithelial neoplasia in human papillomavirus self-sampling of screening non-attenders
Source: Br J Cancer. 2017 Nov 14;118(1):138–44. doi: 10.1038/bjc.2017.371 (PMC5765223; doi:10.1038/bjc.2017.371)
Supplement: Supplementary Table 1 [file bjc2017371x1.docx]

Supplementary table 1: Study characteristics in CSi and Horizon

|  | **CSI** | **HORIZON** |
| --- | --- | --- |
| Population characteristics | Screening non-attenders* residing in Capital Region | Screening samples from routinely screened women |
| Screening area, target population, N | Capital Region, ca. 504,000 | Copenhagen and Frederiksberg municipalities in Capital Region, ca. 218,000 |
| Study period | June 2014-Dec 2015 | Jun 2011-Aug 2011 |
| Study size, N | 23,632 | 5034 |
| Age | 27-65 years | 17-89 years** |
| Collection media | Self-sampling brush, Rovers | LBC, Surepath, BD |
| Preprocessing media | Cervical Brush Diluent (CBD), BD | Surepath, BD |
| HPV assays | Hybrid Capture 2 (HC2, Digene, USA)  CLART® HPV2 (CLART, Genomica, Spain), and  Onclarity (BD, USA). | Hybrid Capture 2 (HC2, Digene, USA)  CLART® HPV2 (CLART, Genomica, Spain), APTIMA (Hologic, USA) and  cobas (Roche Diagnostics, USA). |

* Non-attenders: not been screened the last 4 years (women aged 27-49) or 6 years (women aged 50-65)

** For the analysis in this study, only women aged 27-65 with a primary screening sample were included
